# Determinants of contraceptive decision making among married women in Sub-Saharan Africa from the recent Demographic and Health Survey data

**DOI:** 10.1186/s12905-022-01636-x

**Published:** 2022-02-25

**Authors:** Desalegn Tesfa, Sofonyas Abebaw Tiruneh, Melkalem Mamuye Azanaw, Alemayehu Digssie Gebremariam, Melaku Tadege Engidaw, Mulu Tiruneh, Tsion Dessalegn, Belayneh kefale

**Affiliations:** 1 Department of Public Health, College of Health Sciences, Debre Tabor University, Debre Tabor, Ethiopia; 2 Department of Pharmacy, College of Medicine and Health Science, Bahir Dar University, Bahir Dar, Ethiopia

**Keywords:** Determinants, Contraceptive, Decision making, Married women, Sub-Saharan Africa

## Abstract

**Background:**

Different evidence suggested that couples often disagree about the desirability of pregnancy and the use of contraceptives. Increased women's decision-making on contraceptives is identified as a key solution that can change the prevailing fertility and contraceptive utilization pattern in SSA. Therefore, this study aimed to determine determinants of contraceptive decision-making among married women in Sub-Saharan Africa.

**Methods:**

The data source of this study was the standard demographic and health survey datasets of 33 Sub-Saharan Africa countries. Reproductive age group women aged (15–49 years) currently married who are not pregnant and are current users of contraceptive preceding three years the survey was included from the individual record (IR file) file between 2010 and 2018. Since the outcome variable is composed of polychotomous categorical having multiple-choice, the Multinomial logistic regression (MNLR) model was applied.

**Results:**

A total of 76,516 married women were included in this study. Maternal age 20–35 and 36–49 years were more likely to had decision making on contraceptive use in both women-only and joint (women and husband/partner category (referance = husband/partner) (RRR = 1.2; 95% CI = 1.05–1.41, RRR = 1.18; 1.04–1.33 and RRR = 1.38; 95% CI = 1.17–1.61, RRR = 1.27; 1.11–1.47)] respectively. Married women with higher education were more likely to decide by women-only category on contraceptive use (referance = husband/partner) (RRR = 1.26; 95% CI = 1.06–1.49). Women only decision-making to use contraceptives relative to the husband/partner only decreases by a factor of 0.86 (95% CI = 0.80–0.93) among rural than urban residences. Women only or joint decision making to use contraceptives was 1.25 and 1.35 times more likely relative to husband/partner decision making respectively among women who had work than that of had no work. The relative risk of women's decision to use family planning relative to husband increased among couples who had a marital duration of ≥ 10 years (RRR = 1.14; 95% CI = 1.06–1.22). But it has no significant effect on joint decision making. Respondents found in the richest wealth index category increase the relative risk of joint decision-making relative to husband/partner (RRR = 1.33; 95% CI = 1.20–1.47) compared to the poorest category.

**Conclusions:**

Decision-making to use contraceptives among married women varies greatly by socio-demographic characteristics. The finding of this study showed that women's age, women educational status, residence, duration of the marriage, family economy, and country income were significantly associated with contraceptive decision-making. Therefore to promote ideal family planning decision making, there is a need to formulate policies and design programs that target women's socio-demographic characteristics and modern contraceptive interventions should be promoted by considering empowering women on decision making.

## Background

Family planning is a foremost concern for many developing countries where poor perinatal, intra-natal, postnatal, and child health care services are practiced [1]. The number of children the couples want to have vicissitudes over time [2]. All most all the countries where the fertility rate exceeds five children per woman are in Sub-Saharan Africa (SSA) [3–6]. In the late 1990s, the total fertility rate reached below the replacement level (1.7 per woman) in Europe, northern America, and Australia, consequently, Japan reached below the replacement level in the late 1950s and it has declined further [7, 8]. Increasing women’s decision-making is documented as an important solution that can change prevailing fertility and contraceptive use pattern in SSA [9–12]. Different evidence strongly affirmed that women who are actively involved in household decision-making can control their fertility through the adoption of modern contraception [13].

The Decision-making power of women in family planning is defined as a woman's capability to freely decide independently or discuss with their partner about family planning needs and choices [14]. Contraceptive utilization is commonly compromised by power dynamics between women and her partner as well as those perpetrated by society [9, 15–17]. Decisions about contraceptive use and childbearing may be confounded by unequal power relations [16]. Where couples disagree on fertility preferences or desires, men’s power in a relationship may contribute to greater unmet need and contraception allows women to reduce unwanted, unplanned pregnancies and unsafe abortions [15]. Women have been playing a great role, not only in the enhancement of family well-being but also in the progress of the financial, political social, and ecological atmosphere [18]. Despite the influence that partners may have on decisions, women commonly use family planning covertly, indicating that men and women do not always make decisions as a unit; instead, some women make decisions individually [16, 19].

Women's general participation in decision-making is an important factor in increasing the use of contraceptives [14]. In SSA secret use of contraceptives among women accounts for between 6 and 20% of all contraceptive use [20, 21]. A short interpregnancy interval put endangers the new baby, mother, and previous child [17, 22] (15—2,3). According to the 2015 Global Report, the proportion of reproductive age group women who were married or living in union and used modern contraceptive methods were 57.4% globally, 86% in East Asia, 72% in Latin America, and the Caribbean, 28.5% in Africa and not greater than 22% in SSA [23, 24].

Worldwide, in 2019, 50% of all women of the reproductive age were using some form of contraceptive however, in the same year, Sub-Sahara Africa uses some forms of contraceptive were only 29% [25]. Due to the minimal utilization of contraceptives in Africa, particularly in SSA, women are exposed to unintended pregnancy and as WHO recently reported, around 40% of pregnancy was unplanned [23, 26]. High fertility poses health risks for mothers, children, substantially slow economic growth, and exacerbates environmental degradation [27–30]. As the fertility rate remains high, the youth dependency ratio also increases exponentially [23].

In low and middle-income countries particularly in SSA, continued rapid population growth presents a challenge for achieving sustainable development [25]. Research shows that couples often disagree about the desirability of pregnancy and the use of contraceptives [31]. Therefore, the objective of this study was to assess determinants of contraceptive decision-maker among couples in Sub-Saharan Africa.

## Methods

### Data sources

The study made use of pooled data from current Demographic and Health Surveys (DHS) conducted from 2010 to 2018 among 33 Sub-Saharan Africa countries.. These 33 countries were included in the study because they had current DHS data and also all the variables of interest for this study. Our study included these 33 countries under the DHS program to provide holistic and in-depth evidence of women contraceptive decision making in SSA.

The DHS Program has been working with developing countries around the world to collect data about significant health issues including fertility. This standard Demographic and Health Survey is a population-based survey, nationally representative, contains high-quality data that follow standardized data collection procedures, have consistent content over time, and is collected through uniform questionnaires.

The survey target groups were women aged 15–49 and men aged 15–59 in randomly selected households in each country with a multi-stage stratified cluster sampling design for each country. The study involved a cluster sampling process (i.e. enumeration areas [EAs]), followed by systematic household sampling within the selected EAs. The sample frame usually excludes nomadic and institutional groups such as prisoners and hotel occupants. Detailed information was collected on the background characteristics of the respondents including maternal health and child health [32]. The data for this study were extracted from the individual record (IR file) file from the standard DHS dataset of Sub-Saharan Africa countries with at least one survey from 2010 -2018. A total of 76,516 currently married women who are not pregnant and are current users of family planning were included from 18 low income, 11 lower middle income and 4 upper-middle-income Sub-Saharan countries (11 East African, 6 Central African, 13 West African, and 3 South African countries).

### Eligibility identification

Reproductive age group women aged (15–49 years) currently married women who are not pregnant and are current users of family planning preceding three years the survey in the selected enumeration areas in 33 Sub-Saharan African countries included for this study. Whereas, countries (Central Africa Republic, Eswatini, Sao Tome Principe, Madagascar, and Sudan) did not have a DHS survey report after the 2010/2011 survey year were excluded. As well, three Sub-Saharan Countries (Botswana, Mauritania, and Eritrea) were excluded due to the dataset was not freely available. The outcome variable of this study was the decision-maker to use contraceptives.

### Dependent variables

The dependent variable had three (3) categories namely: the women-only decision making (coded as 1), joint (mother and husband/partner) decision making (coded as 2), and husband/partner-only decision making on contraceptive utilization (coded as 3).

### Statistical analysis

The analysis began with the computation of contraceptive decision-making among married women from 33 Sub Sharan Africa countries. Secondly, we appended the dataset and this generated a total sample of 76,516. After appending, we compute v005/1,000,000 (Women’s individual sample weight/1,000,000) to develop weighted country-based and socio-demographic characteristics (Tables 1, 2).

**Table 1:** Country-based unweighted and weighted samples in SSA using the recent (2010–2018) Demographic and Health Survey data, 2021

Country	Year of DHS	Frequency		Unweighted percent
		Unweighted	Weighted	
*East Africa region*				
Burundi	2016/17	2673	2778	3.49
Ethiopia	2016	2890	3557	3.79
Kenya	2014	4569	4932	5.97
Comoros	2012	3632	3690	4.75
Malawi	2015/16	9286	9385	12.14
Mozambique	2011	1200	1009	1.57
Rwanda	2014/15	3625	3666	4.75
Tanzania	2015/16	2779	3015	3.63
Uganda	2016	4160	4258	5.44
Zambia	2018	3632	3690	4.75
Zimbabwe	2015	3858	3910	5.04
*Central Africa region*				
Angola	2015/16	780	1059	1.02
Democratic Republic of Congo	2013/14	2041	2355	2.67
Republic of Congo	2011/12	2691	2625	3.52
Cameroon	2011	2131	2123	2.79
Gabon	2012	1172	1227	1.53
Chad	2014/15	509	690	0.67
*South Africa region*				
Lesotho	2014	2003	2021	2.62
Namibia	2013	1770	1645	2.31
South Africa	2016	1459	1605	1.91
*West Africa region*				
Burkina Faso	2010	2158	2134	2.82
Benin	2017/18	1694	1684	2.21
Ivory Coast	2011/12	1014	1057	1.33
Ghana	2014	1384	1382	1.81
Gambia	2013	523	562	0.68
Guinea	2018	741	827	0.97
Liberia	2013	1081	1044	1.41
Mali	2018	1293	1456	1.69
Nigeria	2018	4460	4774	5.83
Niger	2012	1288	1146	1.68
Sierra Leone	2013	1695	1683	2.22
Senegal	2010/2011	1123	1298	1.47
Togo	2013/14	1202	1195	1.57
Total		76,516	79,482	100.00

**Table 2:** Unweighted and weighted percentage distribution of selected characteristics

Characteristics	unweighted	Weighted	Unweighted Percent
*Maternal age*			
15–19	2870	2883	3.8
20–35	49,383	51,859	64.5
36–49	24,263	24,740	31.7
*Maternal education*			
No education	15,074	15,908	20.0
Primary	31,898	32,134	40.4
Secondary	24,928	26,294	33.1
Higher	4616	5146	6.5
*Residence*			
Urban	30,464	33,249	41.8
Rural	46,052	46,233	58.2
*Maternal occupation*			
No work	19,422	20,144	25.0
Have worked	57,094	59,698	75.0
*Duration marriage*			
< 10 years	32,477	34,173	43.0
≥ 10 years	44,039	45,309	57.0
*Beating is justifiable*			
No	56,911	59,130	74.4
Yes	19,605	20,352	25.6
*Husband education*			
No education	13,552	14,216	17.9
Primary	26,602	27,272	34.3
Secondary	28,091	29,087	36.6
Higher	8271	8907	11.2
*Husband occupation*			
No work	3808	3907	5.0
Have work	72,708	75,935	95.0
*Family members*			
< 6	38,559	40,743	51.3
6–10	32,571	33,314	41.9
> 10	5386	5436	6.8
*Family income*			
Poorest	12,250	11,085	14.0
Poorer	13,416	13,362	16.8
Middle	15,067	16,039	20.1
Richer	15,937	17,571	22.1
Richest	19,846	21,425	27.0
*Country income*			
Low income	47,361	48,997	61.7
Lower middle income	24,013	25,182	31.6
Upper middle income	5142	5303	6.7
*Sub-regions*			
East Africa	44,345	46,244	58.2
Central Africa	7283	7725	9.7
South Africa	5232	5271	6.6
West Africa	19,656	20,242	25.5
*Reproductive decision making*			
Mmother only	18,640	19,444	24.36
Husband only	7343	7552	9.60
Joint	50,533	52,486	66.04

Multinomial logistic regression (MNLR) model is generally applicable when the outcome variable is composed of polychotomous categorical having multiple choice. It is a simple extension of logistic regression that allows each category of unordered responsive variables to be compared to an arbitrary references category providing several logit regression models.

Multinomial logistic regression models are equivalent to simultaneous estimation of multiple logits where each of the categories is compared to one selected based category.

Let Y_i1_be 1 if the ith decision-maker is manly women-only and 0 otherwise. Similarly, Yi2 be 1 if the ith decision-maker is jointly(women/husband/partner and 0 otherwise. Yi3 be 1 if the ith decision-maker is manly husband/partner and 0 otherwise.

All variables included in bivariate analysis were analyzed in the multinomial logit model. A multinomial logistic regression model was used to estimate variations in the probability of decision-makers to use contraceptives. When using multinomial logistic regression, the relative risk ratios were determined for all independent variables for each category of the dependent variable except the reference category, which is omitted from the analysis. The regression model was fitted to the data to explore the association between a set of independent variables explaining the likelihood of decision making on contraceptive a woman decision making on contraceptive as opposed to being in all other categories. The form of the equation fitted to the data was as follows:

1 $$\mathrm{ln}\frac{p({y}_{i}=m)}{p({y}_{i}=1)}=\mathrm{a}+{\sum }_{k=1}^{k}{\beta }_{xm }{x}_{ik}={z}_{mi}$$

A dependent variable (contraceptive decision-making) that had 3 categories, is represented by m in the equation above, and this requires the calculations for (m-1) equations, one for each category relative to the reference category to describe the likelihood of contraceptive decision making and the independent variables. For the women-only category of the dependent variable, for example, the following equation derived from the latter is then estimated:

2 $$\mathrm{p}({y}_{i}=\mathrm{m})=\frac{\mathrm{exp}\left({z}_{mi}\right)}{1+{\sum }_{n=1}^{m}{exp}^{{(z}_{hi})}}$$

In the multinomial logistic regression, the husband-only is the comparison category. The model parameter estimates and the attendant Relative Risk Ratios (RRR) for the multinomial logit model is that for a unit change in the predictor variable, the logit of outcome m relative to the reference group is expected to change by its respective parameter estimate given that the variables in the model are held constant. The RRRs can be obtained by exponentiation of the multinomial logit coefficients (${e}^{\mathbf{c}\mathbf{o}\mathbf{e}\mathbf{f}\mathbf{f}\mathbf{i}\mathbf{c}\mathbf{e}\mathbf{n}\mathbf{t}}$), or by specifying the **rrr** option. The alpha threshold for significant results was set at p = 0.05 (95%).

## Results

### Weighted and unweighted samples in SSA using the recent Demographic and Health Survey data

Thirty-three(33) SSA countries were included in this study. More than half 42,304 (55.23%) of the respondents were found in the East Africa region with greater than one in ten 9,286 (12.14%) respondents being concentrated in Malawi. Whereas greater than one forth 196, 56 (25.7%) of the participants were found in the West African region with around one in 24 (5.83%) concentrated in Nigeria (Table 1).

### Descriptive characteristics

Around two-third 49,383 (64.5%) of the married women were found in the age category of 20–35 years. Greater than half 46,052 (58.2%) of the married women live in rural areas. There is a somewhat uniform distribution of wealth index. Around three fourth of the respondents were engaged to work and, about half of them belong to the household member of less than six. Three in five 47,361 (61.7%) of the married women were living in a low-income country. About 58.2% of the respondents were from the East Africa Sub-region of SSA. Finally, around two-third (66%) of decision-makers to use contraceptives were jointly (both mother and husband/partner) (Table 2).

### Bivariate analysis

Table 3 shows the bivariate relationship between some selected explanatory variables. Current maternal age, residence, maternal occupation, duration of the marriage, economic status of the family, and country were significantly associated (p < 0.05) with the outcome variable (Table 3).

**Table 3:** Weighted percentage of selected characteristics and contraceptive decision-making using the recent DHS data in SSA, 2021

Characteristics	Decision-maker to use contraceptive			Total	Significant
	Mother only	Husband only	Joint		
*Maternal age*					
15–19	636	333	1914	2883	0.00
20–35	12,318	4991	34,551	51,859	
36–49	6491	2228	16,020	24,740	
*Maternal educational*					
No education	5003	1889	9016	15,908	
Praimary	6848	2859	22,427	32,134	0.00
Secondary	6462	2421	17,411	26,294	
Higher	1131	383	3632	5146	
*Residence*					
Urban	9178	3169	20,902	33,249	0.00
Rural	10,266	4383	31,584	46,233	
*Maternal occupation*					
No worke	5134	2469	12,541	20,144	0.00
Have worke	14,663	5569	39,466	59,698	
*Duration of marriage*					
< 10 years	7714	3304	23,154	34,172	0.00
≥ 10 years	11,731	4248	29,331	45,310	
*Beating is justifiable*					
No	13,336	5127	38,448	59,490	0.09
Yes	5516	2241	12,595	20,352	
*Husband educational status*					
No education	4819	1569	7828	14,216	
Primary	5606	2370	19,296	27,272	0.08
Secondary	7031	2823	19,233	29,087	
Higher	1979	806	6122	8907	
*Husband occupation*					
No work	956	494	2457	3907	0.31
Have work	18,617	7401	49,917	75,935	
*Family members*					
< 6	9510	3608	27,614	40,743	
6–10	8093	3224	21,997	33,314	0.6
> 10	1842	720	2874	5436	
*Family income*					
Poorest	2676	1132	7277	11,085	
Poorer	3201	1338	8823	13,362	
Middle	3798	1583	10,658	16,039	0.00
Richer	4549	1697	11,325	17,571	
Richest	5221	1803	14,401	21,425	
*Country income*					
Low income	10,801	4386	33,810	48,997	
Lower middle income	6443	2519	16,220	25,182	0.00
Upper middle income	2200	646	2457	5303	
*Sub-regions*					
East Africa	8731	3538	33,975	46,244	
Centeral africa	2195	856	4674	7725	0.20
South africa	1593	557	3121	5271	
West Africa	6925	2600	10,717	20,242	

### Multivariate results

Table 4 represents the results of multinomial regression procedures. Maternal age significantly influenced contraceptive decision-making by women-only and jointly (mother and husband). Maternal age 20–35 and 36–49 years increase the relative risk of contraceptive decision making in both mothers only and joint category (relative to the risk husband category) (RRR = 1.22; 95% CI = 1.06–1.41, RRR = 1.18; 1.04–1.33 and RRR = 1.38; 95% CI = 1.18–1.62, RRR = 1.27; 1.11–1.47)] respectively.

**Table 4:** Multinomial regression model predicting the relative risk ratio of deciding to use contraceptive in SSA, 2021

Characteristics	Decision-maker to use contraceptive	
	Women-only RRR 95% CI	Joint (women&husband/partner) RRR 95% CI
*Maternal age*		
15–19	1	1
20–35	*1.22 (1.06–1.41)	*1.18 (1.04–1.33)
36–49	*1.38 (1.18–1.62)	*1.27 (1.11–1.47)
*Maternal educational*		
No education	1	1
Praimary	1.01 (0.93–1.10)	*1.18 (1.10–1.27)
Secondary	1.06 (0.96–1.17)	*1.30 (1.19–1.42)
Higher	*1.26 (1.06–1.50)	*1.62 (1.38–1.90)
*Risidence*		
Urban	1	1
Rural	0.86 (0.80–0.93)	*1.08 (1.01–1.16)
*Maternal occupation*		
No work	1	1
Have work	*1.25 (1.18–1.34)	*1.35 (1.28–1.44)
*Duration of marriage*		
< 10 years	1	
≥ 10 years	*1.14 (1.06–1.22)	0.98 (0.92–1.05)
*Family income*		
Poorest	1	1
Poorer	1.03 (0.94–1.14)	1.05 (0.96–1.14)
Middle	1.03 (0.94–1.14)	1.07 (0.98–1.16)
Richer	*1.10 (1.01–1.21)	1.08 (0.99–1.18)
Richest	*1.28 (1.14–1.43)	*1.33 (1.20–1.47)
*Country income*		
Low income	1	1
Lower middle income	1.11 (1.04–1.20)	0.95 (0.89–1.01)
Upper middle income	*1.83 (1.57–2.12)	*0.68 (0.59–0.79)

* showes *p*-value < 0.05

Holding other variables constant, having higher maternal education increases the relative risk of contraceptive decision-making among mother only category (RRR = 1.26; 95% CI = 1.06–1.50). However in the joint category the relative risk of deciding to use family planning increases starting from primary education up to higher education. The relative risk of women deciding to use contraceptives relative to husbands decreased by a factor of 0.86 (95% CI = 0.80–0.93) among rural residences than urban. However, the relative risk of joint decision to use family planning relative to husband increase by a factor of 1.08; 95% CI = 1.01–1.16) among rural inhabitants as compared to urban. The relative risk of women or joint decision to use family planning relative to husband decision making power category was 1.25 and 1.35 times that of had no work respectively.

The relative risk of women deciding to use contraceptives relative to the husband only increase among couples who had a marital duration of ≥ 10 years (RRR = 1.14; 95% CI = 1.06–1.22). But it has no significant effect in jointly decision-making to use family planning. The richer and the richest family were at a greater risk of falling into the women decide to use family planning category and at less risk of falling into the husband category than the poorest (RRR = 1.10; 95% CI = 1.01–1.21 and RRR = 1.28; 95% CI = 1.14–1.43) respectively.

However keep other variables constant, married women found in the richest wealth index category increase the relative risk of joint decision to use family planning relative to husband (RRR = 1.33; 95% CI = 1.20–1.47) compared to poorest. The relative risk of women and joint decision to use family planning relative to the husband only increase by a factor of 1.83 (95% CI = 1.57–2.12) and 0.68 (95% CI = 0.59–0.79) respectively among respondents found in upper-middle-income countries.

## Discussion

Gender-based power inequalities can contribute to poor health outcomes, for example, hindering communication between partners about reproductive health decision making, constraining women to access to reproductive health survises and by women's and men's attainment to sexual health and pleasure [33]. Throughout resource constraint countries, a considerable proportion of women who do not want to become pregnant were not using contraception. The reason for this “unmet need” for contraception could be women facing multiple barriers to using contraception.

Similarly, in some regions of Africa, there are also noticeable differences.

Although women's empowerment is a key to using contraceptives [19], most partners give the inferior position to women in all aspects of decision-making in developing countries [34]. Besides, little is known about determinants to decision-making to use contraceptives among married women in Sub-Sahara African countries. This study found out that the maternal age group from 20–35 and 36–49 years were significantly more likely to decide for using contraceptives by mothers only and joint (mother and husband/partner) compared to age less than 20 years. This finding affirmed the study was done in [35–38] that showed women with increasing age tend to be gate higher decision-making power for using contraception.

Women with higher education were significantly more likely to have contraceptive decision-making by mothers only compared to those who had no education. However joint (mother and husband/partner decision-making power for using contraceptives were significantly more likely among women who attended primary, secondary and higher education compared that of non-educated. This is consistent with the previous studies [36, 39].

The possible justification is, education empowers women to be independent and equips them with the essential information that may important for deciding their reproductive health issues. In addition, educated women were undergone collective decision-making with their husbands regarding their health care, child health care, and visiting family members or relatives which is important to share experiences and exercise their human and reproductive rights. Studies suggested that greater gender equality may encourage women's autonomy and may facilitate the uptake of contraceptives because of increased female participation in decision-making power [40].

Women only decision making to use contraceptives relative to husband/parent only decision-making power decrease among rural residences than urban counterparts. However, jointly decision making to use contraceptives relative to husband/partner only decision-making increase among rural inhabitants as compared to urban. Studies suggested that geographic variation in the utilization of family planning methods is influenced by several factors like cultural beliefs such as values attached to male dominancy [41] and the presence and quality of reproductive health survises [42]. Similar evidence has been reported from Hundurance [43] and the study conducted in [44] showed that urban women were more likely to make the decision on contraceptive use than rural inhabitants. The possible justification could be urban societies are egalitarian and in contrast, rural societies are patriarchal.

So in a patriarchal society, the majority of the decision including contraceptive utilization has been taken by a husband due to women's economic dependency and existing non-beneficiary traditional culture, however, a joint decision is also practiced in this community instead of mather alone. This study demonstrated that women who had been in marital union for ten or more years were more likely to have women-only decision-making to use contraceptives (relative to the husband-only decision-making power) as compared to those who had been in marital union for less than ten years. This was consistent with a study conducted in [45, 46]. This might be due to the time that partners have spent together has been shown to correlate with marital satisfaction, increase effective communication in marriage often remains impressive, and develop conflict resolution strategies.

Constraint economic resources can inhibit the ability of women to decide on their human and reproductive rights including contraceptive decision-making. likewise in this study, the richer and richest households increase the likelihood of women-only decision-making to use contraceptives (relative to husbands/ partner decision making as compared to those who found in the poorest wealth classification. Similarly, women found in the richest households were had an increased relative risk of joint decision-making to use family planning (relative to husband /partner only decision making to use family planning) compared to the poorest household wealth status.

Inadation women live in the upper-middle-income country increase the likelihood of women decision making to use family planning in relative husband only decision making power as compared to the low-income country. However, the couple's found in upper-middle-income countries decrease the likelihood of joint decision-making to use family planning relative to the husband only compared to in low middle-income countries. Because development and poverty are the most determinants of human and reproductive rights including reproductive decision-making power. Apart from this numerous cultural practices regarding reproductive health decision-making power are associated with countries' development [47] and a challenge for the economy and even political growth. Evidence also supports this finding that women's ability to make household decision-making is enhanced while they are working. Their economic condition stops them from making or even daily household purchases.

The relation between women's empowerment and autonomy in decision-making appears straightforward. Women who had worked were significantly more likely to report to participate in the decision-making process compared to those women who had not been exposed to work [48]. Partner involvement in obstetric decision-making improves the uptake of maternal health services. In addition, in this study, the overall estimate of decision-making to use contraceptives for women only, husband/partner only, and joint (women and spouse) were 24.36%, 9.6%, and 66.04% respectively. A study conducted in Senegal showed that only 6.26% of women have the opportunity to decide on their health. In contrast, 80.33% and 9.66% of their health decider were husband/ partner and jointly respectively [49]. Elsewhere, in several developing countries, studies have shown how certain cultural norms affect women's autonomy in deciding their health [50].

## Conclusions

decision making to use contraceptives among women varies greatly by demographic and socio-economic characteristics. The finding of this study showed that maternal age, maternal educational status, residence, duration of the marriage, family economic status, and country income were significantly associated with contraceptive decision making. Therefore, to promote ideal contraceptive decision making, there is a need to formulate policies and design programs that target women with low education, and Earlier marital age. Attention should be paid to women found in low-income families and low-income countries. Modern contraceptive interventions should be promoted by considering empowering women in decision-making.

## Limitation

There may be threats of internal and external validity because some of the observations were dropped during data cleaning since the study considered currently married women who are not pregnant and are current users of family planning. Therefore the study may be suffered from selection bias. Caution is needed while generalizing this study since it focused on only currently married women who are not pregnant and are current users of family planning. Nevertheless, since we were used the national representative dataset in SSA, the finding could be a true representative for this group of women.

## Data Availability

All the data sets are available on the hand of the corresponding author.

## Abbreviations

DHS: Demographic and Health Survey
MNLR: Multinomial logistic regression
RRR: Relative risk ratio
SSA: Sub Saharan Africa
WHO: World Health Organization
